# An Antibiotic Stewardship Program in Pancreatic Surgery

**DOI:** 10.1001/jamanetworkopen.2025.20149

**Published:** 2025-07-11

**Authors:** Matteo De Pastena, Salvatore Paiella, Erica Secchettin, Damiano Caputo, Luca Moraldi, Danila Azzolina, Laura Addari, Elena Carrara, Anna Maria Azzini, Luca Tirloni, Roberto Coppola, Ilenia Bartolini, Vincenzo La Vaccara, Matteo Risaliti, Roberto Cammarata, Irene Urciuoli, Tommaso Giani, Alessandro Esposito, Luca Casetti, Luca Landoni, Antonio Pea, Martina Fontana, Giuseppe Malleo, Annarita Mazzariol, Dario Gregori, Silvia Angeletti, Massimo Ciccozzi, Carlotta Fiammenghi, Evelina Tacconelli, Roberto Salvia

**Affiliations:** 1 Pancreatic Surgery Unit, University of Verona Hospital Trust, Verona, Italy; 2 University of Verona, Verona, Italy; 3 Pancreatic Surgery Unit, Department of Surgery, Dentistry, Paediatrics and Gynaecology, University of Verona, Verona, Italy; 4 Department of Surgery, Dentistry, Paediatrics and Gynaecology, University of Verona, Verona, Italy; 5 Department of General Surgery, Fondazione Policlinico Universitario Campus Bio-Medico, Rome, Italy; 6 General Surgery Research Unit, University Campus Bio-Medico di Roma, Rome, Italy; 7 Hepatobiliopancreatic Surgery Unit, Careggi University Hospital, Florence, Italy; 8 Department of Environmental and Preventive Science, University of Ferrara, Ferrara, Italy; 9 Division of Infectious Diseases, Department of Diagnostic and Public Health, University of Verona, Verona, Italy; 10 Department of Experimental and Clinical Medicine, University of Florence, Florence, Italy; 11 Department of Surgical Sciences, University of Rome “La Sapienza,” Rome, Italy; 12 Clinical Microbiology and Virology Unit, Careggi University Hospital, Florence, Italy; 13 Pancreatic Surgery Unit, Department of Engineering for Innovation Medicine, University of Verona, Verona, Italy; 14 Unit of Microbiology, University of Verona Hospital Trust, Verona, Italy; 15 Unit of Biostatistics, Epidemiology and Public Health, Department of Cardiac, Thoracic, Vascular Sciences and Public Health, University of Padua, Padua, Italy; 16 Research Unit of Clinical Laboratory Science, Department of Medicine and Surgery, Università Campus Bio-Medico di Roma, Rome, Italy; 17 Fondazione Policlinico Universitario Campus Bio-Medico, Rome, Italy; 18 Unit of Medical Statistics and Molecular Epidemiology, Università Campus Bio-Medico di Roma, Rome, Italy; 19 Department of Foreign Languages and Literatures, University of Verona, Verona, Italy

## Abstract

**Importance:**

Antimicrobial stewardship (AMS) programs optimize antibiotic use and mitigate antimicrobial resistance. The literature on the efficacy of AMS programs in pancreatic surgery is limited.

**Objective:**

To investigate the association of a multifaceted AMS intervention targeting surgical antibiotic prophylaxis (SAP) with the rate of surgical site infections (SSIs) following pancreatic surgery.

**Design, Setting, and Participants:**

This cross-sectional study was a multicenter, before-and-after analysis conducted at 3 Italian centers. The intervention cohort included adult patients aged 18 years or older who underwent pancreatectomy between January 1, 2020, and December 31, 2022, while the historical cohort included patients from January 1, 2015, to December 31, 2019.

**Exposure:**

A multiprofessional, multidimensional ASM program that included a bundle of interventions and pivoted on preoperative rectal screening for multidrug-resistant bacteria and targeted SAP.

**Main Outcomes and Measures:**

The primary outcomes were SSI incidence and SAP appropriateness, assessed through the coverage rate of rectal and biliary isolates. Data were analyzed using propensity score weighting. Secondary outcomes evaluated were other postoperative outcomes (eg, pancreatic fistula rate, length of stay), antibiotic use, and costs.

**Results:**

A total of 3387 patients (median [IQR] age, 66 [66-73] years; 1788 male [52.8%]) were included, with 1219 in the intervention cohort and 2168 in the historical cohort. After implementing the AMS program, a statistically significant reduction was found in rates of overall (30.1% vs 20.6%), superficial (5.8% vs 2.5%), deep (0.9% vs 0.3%), and organ-space (26.3% vs 19.3%) SSIs. After propensity score weighting, the odds ratios for the estimated mean treatment effect were 0.92 (95% CI, 0.89-0.96) for overall, 0.85 (95% CI, 0.78-0.93) for superficial, and 0.95 (95% CI, 0.92-0.99) for organ-space SSIs. Surgical antibiotic prophylaxis coverage increased significantly for rectal screening (87.2% vs 100%) and biliary bacterial colonization (59.7% vs 68.7%). Complications, infections, length of stay, and antibiotic consumption also decreased, with an overall cost savings of 247 460 euros.

**Conclusions and Relevance:**

These findings suggest that a multifaceted, pancreatic surgery–specific AMS program is associated with decreased rates of SSIs, increased coverage of isolated bacteria, improved clinical outcomes, more judicious antibiotic use, and lower costs.

## Key Points

**Question:**

What is the association between an antimicrobial stewardship (AMS) program and the rate of surgical site infections (SSIs) and coverage of bacterial isolates following pancreatic surgery?

**Findings:**

In this cross-sectional study of 3387 patients, the AMS program was associated with a statistically significant reduction of overall, superficial, deep, and organ-space SSIs and increased coverage of rectal and biliary isolates by the tailored surgical antibiotic prophylaxis introduced.

**Meaning:**

These findings suggest that an AMS program for pancreatic surgery may provide more appropriate antimicrobial coverage of isolates to prevent SSIs.

## Teaser

This cross-sectional study investigates the association of an antimicrobial stewardship program using surgical antibiotic prophylaxis and the rate of surgical site infections after pancreatic surgery.

## Introduction

Antimicrobial stewardship (AMS), as defined by the Infectious Diseases Society of America and the Society for Healthcare Epidemiology of America, optimizes antimicrobial therapy selection, dose, route, and duration.^1^ The aim of AMS is to control the spread of microbial infections and reduce the development of antimicrobial resistance by leveraging key elements, such as training for health care practitioners, antibiotic guidelines provision, surveillance programs, and infection control strategies.^2,3^

The application of AMS has recently attracted considerable interest in surgical departments to improve antibiotic prophylaxis and reduce the incidence of associated surgical site infections (SSIs).^4,5,6,7,8,9^ To this end, the European Society of Clinical Microbiology and Infectious Diseases and the European Committee on Infection Control have recommended an active surveillance program with rectal screening, especially for high-risk operations.^10^ Because personalized surgical antibiotic prophylaxis (SAP) for patients with colonized bacteria has been shown to reduce SSIs in medium acuity operations,^11^ strategies for effective AMS have been implemented in other high-stakes procedures, including pancreatic resections.^12^ Pancreatectomies are associated with high rates of SSIs, which contribute to further morbidity, the need for interventional procedures, failure to rescue, and substantially increased hospital stays and costs.^13,14^ Factors associated with postpancreatectomy SSIs are bile bacterial colonization at the time of preoperative biliary drainage^15,16^ or resident multidrug-resistant (MDR) strains.^17^ Initial evidence has been reported of a correlation among preoperative rectal screening cultures, intraoperative bile cultures, and SSI development,^17^ which constitutes the backbone for a tailored SAP in pancreatic resections.

The aim of this study was to evaluate the association of a multifaceted AMS intervention targeting SAP with 30-day SSI incidence and SAP appropriateness in patients scheduled for elective pancreatectomy. Secondarily, cost-effectiveness, antibiotic use, and clinical outcome analyses were performed.

## Methods

### Study Design

This cross-sectional study was initially designed as a multicenter, time series analysis. The historical cohort constituted the prestudy segment of the time series analysis, covering the 3 years prior to the introduction of the AMS program. The poststudy period was divided into three 12-month segments with monthly time points to assess the differences between the prestudy and poststudy slopes of the time series. However, after the first 6 months, an interim evaluation found that the SSI rate per time point was insufficient to perform the segmented regression analysis of interrupted time series. Consequently, a multicenter, before-and-after analysis was conducted with a propensity score weighting momentum to help balance the 2 cohorts (historical vs intervention), as suggested by the referral statistician. The study was conducted at 3 high-volume centers for pancreatic surgery in Italy: the Unit of Pancreatic Surgery of the University of Verona Pancreas Institute (coordinating center), the Hepato-Bilio-Pancreatic Surgery Unit of the Careggi University Hospital, and the Department of General Surgery of the Campus Bio-Medico University. Each center received local institutional review board approval before the study began. All patients provided written informed consent. The study was registered at ClinicalTrials.gov,^18^ and complies with the Strengthening the Reporting of Observational Studies in Epidemiology (STROBE) reporting guideline.^19^

The intervention cohort was prospectively enrolled from January 1, 2020, to December 31, 2022. It included adult patients aged 18 years or older scheduled to receive elective surgical exploration with resection intent (pancreatoduodenectomy, left-side pancreatectomy with or without splenectomy, or total pancreatectomy with or without splenectomy) for any indication. Patients who underwent unplanned procedures, such as atypical pancreatic resection, bypass palliative operation, or surgical exploration only, were retained in the intervention cohort. The historical cohort consisted of patients who underwent elective pancreatic resection between January 1, 2015, and December 31, 2019. Patients with an American Society of Anesthesiologists score of 4 or higher or who were pregnant or breastfeeding were excluded from both cohorts.

The study hypothesis was that a multifaceted, pancreas-specific AMS program, including universal screening for MDR bacteria with preoperative rectal screening and tailored SAP would be associated with an increased coverage of isolated bacteria and a reduced 30-day rate of SSIs. A secondary objective was to assess other infectious complications, surgical postoperative complications, length of stay, antibiotic usage, and cost-effectiveness.

### Procedure

#### Pancreatic AMS Program

The study intervention was the development of a dedicated AMS program for elective pancreatic surgery. Its creation started in 2015 with review of postoperative infectious outcomes and center-specific microbiological data.^17,20^ A multiprofessional team created a bundle of interventions that was established and continuously implemented without any formal washout phase until 2020. Details on the timeline implementation are provided in eMethods 1 in Supplement 1. The AMS program aligned with the National Ministry of Health recommendations, which were published in the same year.^21^ The participating centers already had a surveillance program for MDR bacteria based on preoperative rectal screening and embedded the AMS program into the well-established Enhanced Recovery After Surgery program following the 2019 guidelines.^22^ The AMS bundles and their dimensions and interventions are presented in eMethods 1 in Supplement 1. The relevant items introduced were multidisciplinary team building, infectious control specialist monitoring of surgical unit activities, patient information and engagement, enhanced infection prevention and control measures, tailored SAP, perioperative surgical interventions and SSI management, and educational outreach.

#### Data Collection, Outcomes, and Definitions

The primary outcome measures were the incidence of SSIs at 30 days after surgery and SAP appropriateness (eMethods 2 in Supplement 1). The latter was determined by analyzing rectal screening (eMethods 3 in Supplement 1) and biliary colonization coverage. The effectiveness of SAP was further assessed by comparing rectal screening data (used for SAP selection) and postoperative cultures and expressed as a categorical parameter with a percentage. Finally, the rectal screening results were compared with the bile culture. Data agreement was categorized as positive (MDR bacteria in both cultures), negative (no MDR bacteria in any culture), or wrong (discrepant culture results). The SSI assessment was prospectively performed daily by trained personnel, adopting the Centers for Disease Control and Prevention definition.^23^ A retrospective reevaluation of the historical cohort’s medical records was performed to assess the appropriateness of the SSI definitions in accordance with the Centers for Disease Control and Prevention criteria. Data were extracted from the paper and electronic medical records. As secondary clinical efficacy measures, the postoperative evaluation included the length of stay and the assessment of major complications (Clavien-Dindo grade ≥3).^24^ Other procedure-specific complications were recorded at the 90th postoperative day and graded according to established international guidelines (eMethods 2 in Supplement 1).

Data on systemic antibiotic usage (Anatomic Therapeutic Chemical Classification System code J01) were collected from the pharmacy databases of 2 of the 3 participating centers (University of Verona and Campus Bio-Medico University) using study trimesters as the unit of analysis. The total grams of antibiotics ordered by the participating units were collected for all antibiotic classes and summed to obtain the overall antibiotic consumption and the antibiotic consumption by the World Health Organization (WHO) Access, Watch, and Reserve classification.^5^ The total amount of grams per class was converted into defined daily doses following the 2023 Anatomical Therapeutic Chemical Classification System/defined daily dose index issued by the WHO. Patient-days were used to normalize ward-level antibiotic consumption data and collected from hospital administrative data.

### Statistical Analysis

Descriptive statistics were used to compare characteristics between the intervention and historical groups. Continuous variables are expressed as a median (IQR) and categorical variables as absolute frequency (relative frequency). Continuous variables were compared using *t* tests or Wilcoxon rank sum tests, where appropriate. Categorical variables were compared using χ^2^ or Fisher exact test, where appropriate. Standardized mean differences (SMDs) and 95% CIs are reported. Missing values, such as the number of biliary procedures, cases of cholangitis, and blood transfusions, are reported only in the historical cohort due to the prospective data collection of the intervention cohort. Given the low percentage of missing data (4.6%), the minimal impact of these variables on study outcomes, and the potential for bias introduced by imputation, the latter was not performed. All tests were 2-sided, and the statistical significance was set at *P* < .05.

Before analyzing the SSI occurrence risk rate evolution, propensity score weighting was performed to balance the baseline patient characteristics between the 2 study groups, controlling for the following potential confounding factors associated with group assignment in an observational study framework: age, sex, body max index, Charlson Comorbidity Index, American Society of Anesthesiologists score, presence of jaundice, type of biliary drain, neoadjuvant therapy, rectal screening for MDR bacteria, type of operation, surgical approach, operation time, estimated blood loss, and final pathology (eFigure 1 in Supplement 1). The rationale for including these covariates was based on the following considerations: their potential association with the AMS program (exposure), their possible association with SSI incidence (outcome), and evidence from the literature and clinical expertise. To reduce the impact of extreme values and decrease overlap between cohorts, we applied trimming by excluding propensity score weights above the 95th percentile. The time effect on the AMS program was controlled by estimating a continuous propensity score. The performance of the propensity score balance assessment was visually presented by plotting Spearman correlation of covariates with time before and after the propensity adjustment. The propensity score weighting was estimated via a generalized linear model with inverse probability treatment weights. Among the estimands, the mean treatment effect was selected as the most appropriate and crucial to the unbiased estimation of causal effects and weights. The mean treatment effect was calculated and estimated for the primary outcome to compare differences between treated and untreated groups.

The hypothesis of any intervention association with antibiotic consumption was tested by comparing the preintervention and postintervention phases through a single-group interrupted time series analysis. The development of the AMS program marked the beginning of the intervention. Standard parameters defining the interrupted time series analysis were used to evaluate the hypothetical effect of the antibiotic stewardship intervention on reducing antibiotic consumption. The change in level was used to present the immediate impact of the intervention, while the postintervention trend illustrated the trend of antibiotic consumption during the intervention phase. The trend difference assessed antibiotic consumption changes before and after the intervention. To compare the direct costs of antibiotics (drug consumption of systemic antibiotics presented as the Anatomical Therapeutic Chemical Classification System/Defined Daily Dose index normalized per 1000 patient-days) and evaluate the appropriateness of antibiotic usage, a segmented regression analysis of interrupted time series was performed, focusing on centers with available data. The model was fitted using ordinary least squares regression analysis, and autocorrelation in the error distribution was tested using the Cumby and Huizinga general test for autocorrelation. Changes in slope and level before and after the intervention were assessed using ordinary least squares regression analysis. Statistical analyses were performed using R, version 3.2.2 (R Foundation for Statistical Computing) and Stata, version 14.0 (StataCorp LLC).

## Results

The cohort included 3387 patients who underwent surgical procedures (median [IQR] age, 66 [66-73] years; 1599 female [47.2%] and 1788 male [52.8%]) (Table 1). The study flowchart is presented in eFigure 2 in Supplement 1. The historical cohort comprised 2168 patients (64%) and the intervention cohort, 1219 patients (36%). The adherence rates to the AMS program consistently exceeded 90% across all 3 centers throughout the intervention period. Details on the specific audit parameters are included in eTable 1 in Supplement 1.

**Table 1.** Population Characteristics and Comparisons Between the Historical and Intervention Cohorts

Characteristic	Patients, No. (%)		*P* value	SMD (95% CI)
	Historical cohort (n = 2168)	Intervention cohort (n = 1219)		
**Preoperative**				
Age, median (IQR), y	66 (57 to 72)	66 (57 to 74)	.01	−0.08 (−0.15 to −0.01)
Sex				
Female	972 (44.8)	627 (51.4)	<.001	0.13 (0.06 to 0.20)
Male	1196 (55.2)	592 (48.6)		
BMI, median (IQR)	24.2 (21.8 to 27.0)	24.2 (21.9 to 26.9)	.61	0.00 (−0.07 to 0.07)
Charlson Comorbidity Index ≥4	858 (39.6)	532 (43.6)	.01	0.08 (0.01 to 0.15)
ASA >2	543 (25.0)	319 (26.2)	.33	0.03 (−0.04 to 0.10)
Jaundice	903 (57.6)^a^	470 (54.4)^a^	.12	0.06 (−0.01 to 0.13)
Biliary stenting	819 (52.2)^a^	412 (47.7)^a^	.02	0.08 (0.01 to 0.15)
Multiple biliary procedures	244 (35.1)^b^	126 (30.6)^b^	.14	0.01 (−0.06 to 0.08)
Cholangitis	159 (10.1)^a^	94 (10.9)^a^	.98	0.01 (−0.06 to 0.08)
Neoadjuvant therapy	533 (24.6)	504 (41.3)	<.001	0.36 (0.29 to 0.43)
**Intraoperative**				
Type of surgery			<.001	0.49 (0.42 to 0.57)
Pancreaticoduodenectomy	1319 (60.9)	600 (49.2)		
Distal pancreatectomy	599 (27.6)	355 (29.1)		
Total pancreatectomy	250 (11.5)	139 (11.4)		
Other^c^	NA	125 (10.3)		
Minimally invasive	240 (11.1)	158 (13.0)	.24	0.06 (−0.01 to 0.14)
Vascular resection	337 (15.5)	164 (13.5)	.14	0.06 (−0.01 to 0.13)
Surgery duration, median (IQR), min	390 (310 to 470)	352 (253 to 452)	<.001	0.26 (0.19 to 0.33)
Estimated blood loss, median (IQR), mL	350 (200 to 550)	450 (200 to 800)	<.001	−0.22 (−0.30 to −0.14)
**Pathology**				
Pancreatic ductal adenocarcinoma	1301 (60)	719 (59)	.04	0.18 (0.11 to 0.25)
Pancreatic neuroendocrine tumor	217 (10)	158 (13)		
Intraductal papillary mucinous neoplasm	173 (8)	98 (8)		
Mucinous or serous cystic neoplasm	87 (4)	37 (3)		
Biliary or ampullary cancer	172 (8)	85 (7)		
Other	218 (10)	122 (10)		

Abbreviations: ASA, American Society of Anesthesiologists; BMI, body mass index (calculated as weight in kilograms divided by height in meters squared); NA, not available; SMD, standardized mean difference.

^a^ Patients with pancreatic head lesion.

^b^ Patients with biliary stent.

^c^ Including, for example, pancreatic enucleation, central pancreatectomy, and explorative laparotomy with or without cholecystectomy for intraoperative finding of metastases and local nonresectability.

Table 1 shows preoperative, intraoperative, and postoperative data. The groups differed significantly in age (SMD, −0.08; 95% CI, −0.15 to −0.01; *P* = .01), sex (SMD, 0.13; 95% CI, 0.06-0.20; *P* < .001), and comorbidities (SMD, 0.08; 95% CI, 0.01-0.15; *P* = .01). In the intervention cohort, a higher rate of neoadjuvant therapy was reported (41.3% vs 24.6%; *P* < .001). Higher rates of pancreatoduodenectomies were performed in the historical cohort (60.9% vs 49.2%; *P* < .001). Differences were found in surgery duration (SMD, 0.26; 95% CI, 0.19-0.33; *P* < .001) and estimated blood loss (SMD, −0.22; 95% CI, −0.30 to −0.14; *P* < .001). Blood transfusions did not differ between the groups.

Table 2 details the microbiological data. All 1219 patients in the intervention cohort received rectal screening, compared with 1897 of 2168 patients (87.5%) in the historical cohort (*P* < .001). The rate of bacterial colonization was lower in the intervention group (10.7% vs 15%; *P* < .001). The rate of colonization of the biliary tree was similar between the 2 groups (60.6% vs 61.4%; *P* = .31).

**Table 2.** Microbiological Data Comparison

Variable	Patients, No. (%)		*P* value	SMD (95% CI)
	Historical cohort (n = 2168)	Intervention cohort (n = 1219)		
Positive rectal screening	285 (15.0)^a^	130 (10.7)	<.001	0.55 (0.48-0.63)
Vancomycin-resistant enterococci	18 (0.9)	18 (1.5)		
ESBL-producing Enterobacteriaceae	254 (13.4)	105 (8.5)		
Carbapenem-resistant Enterobacteriaceae	36 (1.9)	17 (1.4)		
Carbapenem-resistant *Klebsiella pneumoniae*	31 (1.6)	15 (1.2)		
Carbapenem-resistant *Pseudomonas*	2 (0.1)	0 (0)		
Carbapenem-resistant *Acinetobacter*	2 (0.1)	1 (0.1)		
Positive bile culture	742 (61.4)^b^	413 (60.6)^c^	.31	0.19 (0.12-0.26)
Vancomycin-resistant enterococci	14 (1.2)	14 (2.1)		
ESBL-producing Enterobacteriaceae	154 (12.7)	72 (10.6)		
Carbapenem-resistant Enterobacteriaceae	51 (4.2)	22 (3.2)		
Carbapenem-resistant *Klebsiella pneumoniae*	19 (1.6)	11 (1.6)		
Carbapenem-resistant *Pseudomonas*	6 (0.5)	5 (0.7)		
Carbapenem-resistant *Acinetobacter*	1 (0.1)	0 (0)		
Accordance rectal screening, bile culture	995 (82.3)^b^	547 (80.3)^c^	.22	0.25 (0.18-0.32)
SAP covering rectal screening isolate	1652 (87.2)^a^	1219 (100)	<.001	0.19 (0.12-0.26)
SAP covering bile contamination	721 (59.7)^b^	468 (68.7)^c^	<.001	0.19 (0.12-0.26)
Accordance rectal screening, MDR gram-negative sustained superficial SSIs	76 (60.3)^d^	25 (80.6)^d^	<.001	0.65 (0.30-1.0)
Accordance rectal screening, MDR gram-negative sustained organ-space SSIs	379 (66.4)^d^	162 (68.9)^d^	<.001	0.46 (0.31-0.62)
Accordance bile culture, MDR gram-negative sustained superficial SSIs	33 (26.4)^d^	8 (25.8)^d^	.57	0.35 (0.0-0.70)
Accordance bile culture, MDR gram-negative sustained organ-space SSIs	88 (15.4)^d^	38 (16.2)^d^	<.001	0.37 (0.22-0.52)

Abbreviations: ESBL, extended-spectrum β-lactamase; MDR, multidrug resistant; SAP, surgical antibiotic prophylaxis; SSI, surgical site infection; SMD, standardized mean difference.

^a^ Of 1895 patients, 237 (12.5%) did not receive the rectal swab; the total exceeds 100% given that multiple bacteria could be present.

^b^ A total of 1208 patients (pancreaticoduodenectomy and total pancreatectomy).

^c^ A total of 681 patients (pancreaticoduodenectomy and total pancreatectomy).

Regarding the primary outcome, the intervention vs historical group showed statistically significant rate reductions in overall (20.6% vs 30.1%; *P* < .001), superficial (2.5% vs 5.8%; *P* < .001), deep (0.3% vs 0.9%, *P* = .05), and organ-space (19.3% vs 26.3%, *P* < .001) SSIs (Table 3). Microbiological data of bacteria-sustaining SSIs are reported in eFigures 3 to 5 in Supplement 1.

**Table 3.** Primary Outcome Analysis

Surgical site infection	Patients, No. (%)		Before propensity score weighting		After propensity score weighting	
	Historical cohort (n = 2168)	Intervention cohort (n = 1219)	*P* value	SMD (95% CI)	*P* value	OR (95% CI)
Overall	653 (30.1)	251 (20.6)	<.001	0.21 (0.14-0.28)	NA	NA
**Superficial**						
All	126 (5.8)	31 (2.5)	<.001	0.16 (0.09-0.23)	.008	0.85 (0.75-0.96)
Vancomycin-resistant enterococci	6 (4.8)	0 (0)				
ESBL-producing Enterobacteriaceae	24 (19)	1 (3.2)				
Carbapenem-resistant Enterobacteriaceae	10 (8)	2 (6.5)				
Carbapenem-resistant *Klebsiella pneumoniae*	4 (3.2)	1 (3.2)				
Carbapenem-resistant *Pseudomonas*	1 (0.8)	0 (0)				
**Deep**						
All	19 (0.9)	4 (0.3)	.05	0.07 (0.0-0.14)	.05	0.80 (0.63-1.0)
Vancomycin-resistant enterococci	1 (5.3)	0 (0)				
ESBL-producing Enterobacteriaceae	6 (31.6)	1 (25)				
Carbapenem-resistant Enterobacteriaceae	1 (5.3)	0 (0)				
Carbapenem-resistant *Klebsiella pneumoniae*	1 (5.3)	0 (0)				
Carbapenem-resistant *Pseudomonas*	0 (0)	0 (0)				
**Organ-space**						
All	571 (26.3)	235 (19.3)	<.001	0.17 (0.10-0.24)	.04	0.87 (0.75-0.99)
Vancomycin-resistant enterococci	18 (3.2)	12 (5.1)				
ESBL-producing Enterobacteriaceae	110 (19.3)	45 (19.1)				
Carbapenem-resistant Enterobacteriaceae	58 (10.2)	13 (5.5)				
Carbapenem-resistant *Klebsiella pneumoniae*	37 (6.5)	8 (3.4)				
Carbapenem-resistant *Pseudomonas*	13 (2.3)	0 (0)				
Carbapenem-resistant *Acinetobacter*	6 (1.1)	0 (0)				

Abbreviations: ESBL, extended-spectrum β-lactamase; NA, not applicable; OR, odds ratio; SMD, standardized mean difference.

The results of the primary and secondary outcomes based on center-specific data and the intervention cohort, which included only standard pancreatic resection, are detailed in eTables 2 and 3 in Supplement 1. After propensity score weighting, the reduction of the primary outcome was confirmed. The odds ratio for the estimated mean treatment effect was 0.92 (95% CI, 0.89-0.96; *P* < .001) for overall SSIs, 0.85 (95% CI, 0.78-0.93; *P* < .001) for superficial SSIs, and 0.95 (95% CI, 0.92-0.99; *P* = .04) for organ-space SSIs. Additionally, the estimated probability of developing an SSI over time is reported in eFigure 6 in Supplement 1. Surgical antibiotic prophylaxis coverage increased significantly from the pre- to the postintervention period for rectal screening and biliary colonization (87.2% vs 100% and 59.7% vs 68.7%, respectively) (*P* < .001). Tailored SAP was significantly associated with better coverage for superficial and organ-space SSIs caused by MDR, gram-negative bacteria than broad-spectrum antibiotics, with coverage rates of 58.1% and 63.4% vs 32.3% and 34.7%, respectively (*P* < .001). During the study period, rectal screening showed similar accuracy in estimating the presence of MDR bacteria in biliary culture (80.3% vs 82.3%; *P* = .22). The SSI rate in patients with discordance between rectal screening and biliary culture was 6.4%, with only 3.2% attributed to MDR bacteria.

Table 4 presents the postoperative outcomes and group comparisons. The intervention vs historical group showed lower overall (49.1% vs 58.1%; *P* < .001) and major (15.8% vs 20%; *P* = .002) complication rates. The intervention group also showed lower rates of hospital-acquired infections (30.1% vs 40.5%; *P* < .001), postoperative pancreatic fistula (18.8% vs 20.5%; *P* < .001), postpancreatectomy hemorrhage (10.5% vs 14.1%; *P* = .001), delayed gastric emptying (10.3% vs 13.0%; *P* = .01), and a shorter length of stay (median [IQR], 9 [7-18] vs 10 [7-18] days; *P* < .001). Regarding postpancreatectomy hemorrhage, 40% of events were associated with postoperative pancreatic fistula grade B or C. These results were partially confirmed after the propensity score weighting, also shown in Table 4.

**Table 4.** Postoperative Course

Postoperative course	Patients, No. (%)		Before propensity score weighting		After propensity score weighting	
	Historical cohort (n = 2168)	Intervention cohort (n = 1219)	*P* value	SMD (95% CI)	*P* value	OR (95% CI)
Any complications	1260 (58.1)	599 (49.1)	<.001	0.18 (0.11 to 0.25)	.001	0.87 (0.80 to 0.95)
Major complications^a^	433 (20.0)	192 (15.8)	.002	0.11 (0.04 to 0.18)	.97	0.99 (0.91 to 1.1)
Hospital-acquired infection (any type)	879 (40.5)	367 (30.1)	<.001	0.22 (0.15 to 0.29)	.03	0.88 (0.80 to 0.98)
Postoperative pancreatic fistula						
Grade B	314 (16.4)	185 (17.1)	<.001	0.11 (0.04 to 0.18)	.81	0.98 (0.88 to 1.1)
Grade C	79 (4.1)	18 (1.7)				
Biliary fistula (any grade	114 (5.3)	83 (6.8)	.11	0.07 (−0.01 to 0.14)	.02	1.1 (1.0 to 1.2)
Chyle leak (any grade)	97 (4.5)	52 (4.3)	.82	0.01 (−0.06 to 0.08)	.33	1.1 (0.94 to 1.2)
Enteric fistula (any grade)	56 (2.6)	27 (2.2)	.57	0.02 (−0.05 to 0.09)	.26	1.1 (0.96 to 1.2)
Postpancreatectomy hemorrhage (any grade)	306 (14.1)	128 (10.5)	.001	0.11 (0.04 to 0.18)	.46	0.96 (0.89 to 1.0)
Delayed gastric emptying (any grade)	282 (13.0)	126 (10.3)	.01	0.08 (0.01 to 0.15)	.33	0.89 (0.73 to 1.1)
Intensive care unit admission (any grade)	262 (12.1)	124 (10.2)	.05	0.06 (−0.01 to 0.13)	.78	0.97 (0.85 to 1.2)
Reoperation	179 (8.3)	84 (6.9)	.15	0.05 (−0.02 to 0.12)	.55	0.97 (0.89 to 1.1)
Length of stay, median (IQR), d	10 (7-18)	9 (7-18)	<.001	0.07 (0.0 to 0.14)	.73	−0.06 (0.44 to 0.32)
Mortality	70 (3.2)	35 (2.9)	.34	0.02 (−0.05 to 0.09)	.35	0.94 (0.84 to 1.1)

Abbreviations: OR, odds ratio; SMD, standardized mean difference.

^a^ Clavien-Dindo classification^24^ grade of 3 or more.

Regarding the evaluation of antibiotic use and related cost-effectiveness analysis, the interrupted time series analysis revealed a statistically significant reduction in mean (SD) overall antibiotic consumption in the intervention cohort compared with the historical cohort from 753.54 (91.30) to 652.58 (90.82). This decrease was observed in specific antibiotic groups, including those in the WHO Watch category, such as meropenem (from a mean [SD] of 76.37 [35.32] to 46.95 [19.51] defined daily doses per patient-day) and ciprofloxacin (from a mean [SD] of 117.40 (32.7) to 22.07 (11.62) defined daily doses per patient-day (full results of the interrupted time series analysis are presented in Table 5). Concurrently, a reduction in the direct costs associated with antibiotic use of 247 460 euros was identified.

**Table 5.** Interrupted Time Series Analysis of Antimicrobial Consumption Data (Defined Daily Doses Per 1000 Patient-Days) by the AWaRE Classification^5^

Variable	Coefficient (SE) [95% CI]					*P* value
	Preintervention trend	Change in level	Change in trend	Starting level	Postintervention trend	
Overall antibiotic consumption	−1.83 (3.43) [−8.87 to 5.21]	−96.85 (54.62) [−208.75 to 15.04]	3.37 (5.95) [−8.83 to 15.58]	767.38 (24.38) [717.43 to 817.32]	1.54 (4.77) [−8.24 to 11.32]	.01
AWaRE category						
Access	0.89 (2.35) [−3.93 to 5.73]	24.64 (38.79) [−54.81 to 104.10]	2.71 (4.45) [−6.41 to 11.84]	201.31 (17.01) [164.62 to 237.99]	3.61 (3.81) [−4.19 to 11.43]	.28
Watch	−2.61 (3.91) [−10.63 to 5.41]	−91.66 (49.71) [−193.49 to 10.16]	0.36 (4.17) [−8.18 to 8.90]	475.16 (23.09) [427.85 to 522.47]	−2.24 (1.47) [−5.25 to 0.76]	<.001
Reserve	0.17 (1.82) [−3.56 to 3.90]	−29.11 (30.72) [−92.06 to 33.82]	0.49 (2.72) [−5.08 to 6.07]	91.02 (15.82) [58.60 to 123.43]	0.66 (2.09) [−3.62 to 4.96]	.59
Antibiotic						
Piperacillin or tazobactam	1.87 (2.02) [−2.26 to 6.01]	−23.74 (29.7) [−84.65 to 37.16]	−0.71 (2.80) [−6.45 to 5.03]	130.40 (10.42) [109.05 to 151.74]	1.16 (1.83) [−2.59 to 4.91]	.63
Meropenem	−2.68 (1.46) [−5.68 to 0.31]	7.57 (17.2) [−27.66 to 42.81]	0.80 (1.78) [−2.85 to 4.46]	96.54 (15.15) [65.49 to 127.59]	−1.88 (1.03) [−4.01 to 0.24]	.004
Ciprofloxacin	−3.93 (1.31) [−6.61 to −1.24]	−52.2 (10.73) [−74.19 to −30.22]	2.64 (1.40) [−0.24 to 5.52]	146.90 (14.51) [117.17 to 176.64]	−1.29 (0.51) [−2.34 to −0.23]	<.001
Antibiotic cost, euros	−1030.95 (523.80) [−2103 to 42.01]	−4891 (6677.83) [−1857.07 to 8788.01]	789.32 (648.02) [−538.09 to 2116.74]	44 323.08 (5713.28) [32 619.96 to 56 026.2]	−241.62 (382.07) [−519.53 to 541.01]	.003

Abbreviation: AWaRE, Access, Watch, Reserve.

## Discussion

This cross-sectional study shows that an AMS program specifically designed for pancreatic surgery was associated with clinical and economic advantages. Following surgical exploration with resection intent for any indications, a statistically significant increase in coverage from isolates from the biliary and gastrointestinal tract was found, confirming the appropriateness of the tailored SAP. In addition, the AMS program implementation was associated with a statistically significant decrease in all SSIs. Finally, the program was associated with minimized key antibiotic consumption, in line with WHO recommendations.^6,7,8,9,25^

The term AMS refers to coordinated activities aimed at optimizing antibiotic prescription and use. These activities include selecting the best drug regimen in terms of dosage, duration, and route of administration while minimizing the spread of MDR bacteria and achieving clinical benefits. Despite its importance, there are few reports on AMS in high-acuity surgery, and none have been specifically developed for pancreatic surgery. Pancreatic surgery presents unique challenges for infectious disease specialists due to several factors, such as preoperative conditions (obstructive jaundice with a biliary stent or cholangitis if tumors are located in the pancreatic head) and postoperative complications (pancreatic fistula, postoperative bleeding, intensive care unit admission). The multifaceted approach applied in this study included several enhanced infection prevention and control measures, such as updating the local policies and guidelines, universal rectal screening surveillance, prevalence reports, education, and practice audits. A key component of this program was the introduction of universal rectal screening in clinical practice. Rectal screening became the foundation of the active surveillance program, which allowed for the rapid identification of patients with bacteria colonization. These patients were flagged with isolation codes, and specific infection control strategies were implemented to reduce the transmission of MDR bacteria within the hospital.

Current guidelines for pancreatic surgery recommend first- or second-generation cephalosporins for SAP. However, these broad-spectrum antibiotics may not be effective for patients at higher risk of infectious complications, such as those with biliary stents who are more likely to carry MDR, gram-negative bacteria.^15,16,17,26,27^ In this framework, the presented AMS program enhances the concept of tailored SAP by addressing specific clinical needs. This approach not only promotes prudent antibiotic use but also leads to substantial cost savings.^28^ Shifting the focus from treating SSIs to controlling MDR bacterial colonization may minimize the use of crucial antibiotics, classified as Watch; encourage the use of more commonly prescribed antibiotics, classified as Access; and reduce unnecessary postoperative doses. This study aligns with the WHO Access, Watch, and Reserve classification and recommendations for antibiotic use, in contrast to recent articles that advocated for increased reliance on broad-spectrum antibiotics, such as piperacillin and tazobactam,^29^ and extended therapy until the third postoperative day.^30^

Valuable insights can be gained from the analysis of microbiological data. First, the preoperative rectal screening was associated with the MDR bacterial colonization in the biliary tract in more than 80% of patients. Second, the tailored SAP was significantly associated with better coverage for both rectal screening and bile MDR, gram-negative contamination compared with broad-spectrum antibiotic use, with rates of 100% vs 87.2% for rectal screening and 68.7% vs 59.7% for bile contamination. Third, the preoperative rectal screening was associated with the presence or absence of MDR bacteria associated with superficial and organ-space SSIs, suggesting a possible cause-and-effect mechanism and reinforcing the idea that these bacteria should be the focus of any antimicrobial interventions. Fourth, tailored SAP was significantly associated with better coverage for superficial and organ-space SSIs caused by MDR, gram-negative bacteria than broad-spectrum antibiotics, with coverage rates of 58.1% and 63.4% vs 32.3% and 34.7%, respectively. The overall clinical benefits observed in the intervention cohort can be understood through the lens of previous research, according to which SSIs can be viewed as a driving factor, rather than as a consequence, of major postoperative complications.^31^

### Limitations

This study had some limitations. First, the study design shifted from a time series to a before-and-after analysis, potentially introducing bias. Propensity score weighting could address some bias in balancing cohorts, but its application was limited to observed characteristics and did not account for unobserved ones, which means that it could not provide a causal interpretation. Second, the overlap with the COVID-19 pandemic may have contributed to the reduction of SSIs due to increased awareness and implementation of infection control measures. Third, the tailored SAP used in this study did not cover infections caused by vancomycin-resistant *Enterococcus* species. Nonetheless, the existing literature does not support the use of tailored prophylaxis for these species or indicates that it is associated with worse postoperative outcomes. Fourth, differences in surgical volume and internal organization among the participating centers may introduce bias. Fifth, the general applicability may be challenging because implementing an AMS program for high-stakes procedures is a complex and time-consuming process that requires close collaboration among multiple specialists, adequate hospital facilities, and robust infection control measures and strategies. Sixth, some novelties were implemented and standardized in clinical practice over the long study period (eg, minimally invasive surgery, surgical drain management). Still, we believe that the individual contribution of these factors may not outweigh the generalized effect of the AMS program.

## Conclusions

This cross-sectional found that a multifaceted, pancreatic surgery–specific AMS program was associated with reduced SSIs, broader coverage of isolated bacteria, and improved clinical outcomes. Furthermore, these findings also suggest that AMS leads to more appropriate antibiotic use and lower costs.

## Notes

Supplement 1.
eMethods 1.
**eTable 1.** AMS Items Embedded Into an Enhanced Recovery After Surgery (ERAS) Model of Perioperative Care
eMethods 2.

eMethods 3.
**eFigure 1.** Propensity Score Weighting Covariate Balance **eFigure 2.** Study Flowchart **eFigure 3.** Microbiological Report of Superficial SSI **eFigure 4.** Microbiological Report of Deep SSI **eFigure 5.** Microbiological Report of Organ and Space SSI **eTable 2.** The Primary Outcome Through Center-Specific Analyses **eTable 3.** Primary and Secondary Outcomes, Including Only Formal Pancreatic Resections **eFigure 6.** Estimated Probabilities of SSI
eReferences.

Supplement 2.
Data Sharing Statement
