# Supplementary material for: An Antibiotic Stewardship Program in Pancreatic Surgery
Source: JAMA Netw Open. 2025 Jul 11;8(7):e2520149. doi: 10.1001/jamanetworkopen.2025.20149 (PMC12254888; doi:10.1001/jamanetworkopen.2025.20149)
Supplement: Supplement 1. — eMethods 1. eTable 1. AMS Items Embedded Into an Enhanced Recovery After Surgery (ERAS) Model of Perioperative Care eMethods 2. eMethods 3. eFigure 1. Propensity Score Weighting Covariate Balance eFigure 2. Study Flowchart eFigure 3. Microbiological Report of Superficial SSI eFigure 4. Microbiological Report of Deep SSI eFigure 5. Microbiological Report of Organ and Space SSI eTable 2. The Primary Outcome Through Center-Specific Analyses eTable 3. Primary and Secondary Outcomes, Including Only Formal Pancreatic Resections eFigure 6. Estimated Probabilities of SSI eReferences. [file jamanetwopen-e2520149-s001.pdf]

Supplemental Online Content

De Pastena M, Paiella S, Secchettin E, et al. An antibiotic stewardship program in pancreatic surgery. *JAMA Netw Open*. 2025;8(7):e2520149.
doi:10.1001/jamanetworkopen.2025.20149

eMethods 1.

eTable 1. AMS Items Embedded Into an Enhanced Recovery After Surgery (ERAS) Model of Perioperative Care

eMethods 2.

eMethods 3.

eFigure 1. Propensity Score Weighting Covariate Balance

eFigure 2. Study Flowchart

eFigure 3. Microbiological Report of Superficial SSI

eFigure 4. Microbiological Report of Deep SSI

eFigure 5. Microbiological Report of Organ and Space SSI

eTable 2. The Primary Outcome Through Center-Specific Analyses

eTable 3. Primary and Secondary Outcomes, Including Only Formal Pancreatic Resections

eFigure 6. Estimated Probabilities of SSI

eReferences.

Supplement 2. **Data Sharing Statement**

This supplemental material has been provided by the authors to give readers additional information about their work.

© 2025 De Pastena M et al. *JAMA Network Open*.

eMethods 1

Multifaceted pancreatic surgery-specific antimicrobial stewardship (AMS) program relevant items:

- *Multidisciplinary team building*. Each center established an interdisciplinary team that included surgeons, infection control specialists, infectious disease specialists, and microbiologists, all serving as referrals for their respective units. The scientific foundation of the AMS program was based on previous research[1-3], along with a systematic review of the literature.
- *Infectious control specialists monitoring surgical unit activities*. Referral infection control specialists monitored the routine clinical activities of each unit for at least three months. They evaluated clinical practice and provided timely interventions as needed.
- *Patient information and engagement*. During the patient interview, investigators collected medical histories and outlined the clinical and surgical perioperative pathways. Those patients identified as having rectal colonization by MDR bacteria were informed that they might be required to undergo contact isolation. Furthermore, a pilot group of isolated patients received psychological evaluation and support as needed during their isolation.
- *Enhanced infection prevention and control measures.* The universal rectal screening was included as part of the routine preoperative tests. As described elsewhere[1], it served as a screening method to detect patients colonized by MDR bacteria, including extended-spectrum beta-lactamases producing Enterobacterales, carbapenem-resistant Enterobacterales, Pseudomonas aeruginosa, Acinetobacter, and Vancomycin-resistant Enterococci. The details of the procedure are reported in eMethods 2. When MDR bacteria were detected on preoperative rectal screening, infection control measures were implemented, including contact precautions. A red flag isolation code was used to ensure prompt and appropriate care, reducing the risk of horizontal transmission. The rectal screening was repeated during the postoperative period if the patient required more than two days of ICU recovery or if the infectious disease specialist deemed it necessary. In accordance with institutional guidelines,

© 2025 De Pastena M et al. *JAMA Network Open*.

patients and caregivers were instructed on measures to minimize the spread of MDR bacteria upon discharge. Additionally, for patients with extended hospital stays, a pilot group received psychological evaluation and support as needed during their isolation.

- *Tailored surgical antibiotic prophylaxis*. The infectious disease specialists determined the optimal antibiotic regimen according to the rectal screening results, prioritizing the avoidance of key antibiotics for all patients. The tailored SAP was reserved for extended-spectrum beta-lactamases producing Enterobacterales, carbapenem-resistant Enterobacterales, Pseudomonas aeruginosa, and Acinetobacter species. To avoid using key antibiotics, the vancomycin-resistant Enterococci were excluded due to the lack of evidence about their impact on the postoperative clinical course. Details of the SAP are provided in the Supplementary Table. The choice of antibiotics was guided primarily by the planned operation, specifically by the need for an enteric anastomosis. The standard SAP consisted of a β-lactam (amoxicillin) combined with a β-lactamase inhibitor (clavulanic acid) for pancreatoduodenectomy and total pancreatectomy. For left pancreatectomy, a first-generation cephalosporin (cefazolin) was used instead. If the rectal screening identified MDR pathogens, such as ESBL bacteria, ertapenem was administered. For other specific carbapenem-resistant bacteria, the infectious diseases specialist prescribed tailored SAP based on the antibiogram from the rectal screening. The SAP was administered as a single preoperative dose. Redosing was considered based on the procedure duration, the antibiotic half-life, and intraoperative blood loss exceeding 1500 cc. The SAP was not continued in the postoperative period. In the historical cohort, the SAP varied throughout the study period, but it was never based on the type of pancreatic resection performed. Furthermore, no SAP adjustment was made based on the rectal screening results. The antibiotics used included ampicillin/sulbactam, amoxicillin/clavulanic acid, cefazolin, and piperacillin/tazobactam during a 16-month experimental study involving patients undergoing pancreatoduodenectomy[4].

© 2025 De Pastena M et al. *JAMA Network Open*.

- *Perioperative surgical interventions and SSI management*. Surgical procedures, drain management protocols, and perioperative care adhered to institutional policies and best practices. Bile cultures were routinely performed intraoperatively for total pancreatectomy and pancreatoduodenectomy. Drain fluid cultures were obtained postoperatively if signs of infection arose, such as purulent drainage, fever, abnormal laboratory tests, or abnormal imaging findings. Locally calibrated SSI management guidelines were developed and implemented across centers, following a stepwise approach. Initial treatment involved antibiotic therapy, always planned in consultation with the infectious disease specialist and adjusted based on the bile culture result (if available). If antibiotic therapy failed, percutaneous or endoscopic drainage was considered. Severe cases were discussed with the infectious disease specialists.
- *Educational outreach*. Healthcare professionals received training from experienced infectious disease specialists and microbiologists through in-person lectures and active workgroups in the ward. Various educational initiatives, including seminars, classes, and workshops, were organized to enhance their knowledge. Additionally, healthcare professionals were educated on the definitions of study outcomes (e.g., SSIs definition) and the relevant metrics. Initially, a quarterly evaluation of two to three random cases was planned for each unit. However, due to the constraints imposed by the COVID-19 pandemic, these evaluations were conducted at least twice a year via online teleconferences. Training courses were developed to focus on the principles of antibiotic use and SAP, including selection, dosage, route of administration, and duration of appropriate antimicrobial therapy. By incorporating insights from all healthcare professionals involved in the study, the multidisciplinary team created the courses. The team also conducted random reviews of patient records to evaluate the efficacy of the local guidelines. Subsequently, through problem-solving strategies, the guidelines were implemented to prevent future errors.

© 2025 De Pastena M et al. *JAMA Network Open*.

eTable 1. AMS Items Embedded Into an Enhanced Recovery After Surgery (ERAS) Model[5] of Perioperative Care

**Preoperatively**	
First visit	• Adequate and correct counseling on the perioperative period<br>• Recording of patient's allergies<br>• Recording of any antibiotic treatments during the last 3 months
Preoperative tests (within two weeks from surgery)	• Universal rectal screening to determinate MDR bacteria colonization
One-to-two days before surgery	• Abdominal hair removal avoiding shaving<br>• Preoperative chlorhexidine skin cleansing the night before and the morning of surgery (e.g., shower, wipes)
**Intraoperatively**	
Staff behavior	• Hand washing and sterile clothing training course for residents and new nurses<br>• Personnel traffic limited to the essential
Antibiotic prophylaxis	• Tailored and based on the rectal screening results (see Table S2)<br>• Optimization of administration and redosing (following the surgery duration and blood loss)
Skin disinfection	• Single-use and sterile chlorhexidine antiseptic disposables
Hypothermia avoidance	• Forced-air warming system and intravenous warming set
Bile sampling	• Bile culture (in pancreaticoduodenectomy and total pancreatectomy)
**Postoperatively**	
Patient Care	• Early mobilization<br>• Early oral feeding<br>• Pulmonary physio-kinetic rehabilitation<br>• Restricted relatives visit (1h/day)
Surgical Care	• No drain placement or early drains removal policy<br>• Antibiotic empiric therapy based on intraoperative bile culture after infectious diseases specialists' consultation

© 2025 De Pastena M et al. *JAMA Network Open*.

Personnel behavior	• Pain control service assistance (twice/day until postoperative day 3; once/day until complete pain control)<br>• Contact isolation of MDR-colonized patients<br>• Repeated rectal screening after ICU stays, or when MDR colonization is suspected<br>• Hands Hygiene (training course for residents and new nurses)<br>• Education and training of the personnel<br>• Gloves and surgical masks in the ward<br>• Gloves changing for each patient<br>• Increased alcohol gels positions in the ward (one in each patient room and in the aisles)

MDR: Multi-Drug Resistant; ICU: intensive care unit.

Supplementary Table. Tailored Antibiotic Prophylaxis Based on Preoperative Rectal Swab

**Type of surgery**	**Antibiotic**
Left pancreatectomy	Cefazolin (Cefamezin®, Pfizer Italia Srl, Rome, Italy) 2 gr IV preoperatively + 1 gr IV after 4h from the first dose (intraoperatively)<br>If allergy:<br>Clindamycin Hikma® (Hikma Pharma GmbH, Gräfelfing, 900 mg IV preoperatively and 600 mg IV after the first dose (intraoperatively)
Pancreaticoduodenectomy and total pancreatectomy	Amoxycillin/Clavulanic acid (Duclav®, Samarth Life Sciences Pvt. Ltd., Mumbai, India) acid 2 gr IV preoperatively + 1 gr IV after 2h from the first dose (intraoperatively);<br>If Allergy:<br>Clindamycin Hikma® (Hikma Pharma GmbH, Gräfelfing, Germany), 900 mg IV preoperatively and 600 mg IV after the first dose (intraoperatively) + Gentamicin (Fisiopharma S.r.l. Palomonte, Salerno, Italy) 5 mg/kg IV preoperatively.
**MDR colonized patients**	
Extended spectrum beta-lactamase (ESBL) - Enterobacterales	Ertapenem (Invanz®, Merck & co., Rahway, New Jersey, USA), 1gr preoperatively.

© 2025 De Pastena M et al. *JAMA Network Open*.

Carbapenem-resistant Enterobacterales (CRE),	Based on the preoperative rectal screening antibiogram.
Carbapenem-resistant *Acinetobacter baumannii,* <br>Carbapenem-resistant *Pseudomonas aeruginosa*	In the emergency setting: Clindamycin Hikma® (Hikma Pharma GmbH, Gräfelfing, Germany), 900 mg IV preoperatively and 600 mg IV after the first dose (intraoperatively) + Gentamicin (Fisiopharma S.r.l. Palomonte, Salerno, Italy) 5 mg/kg/dose preoperatively.

Infection Control measures adopted during the historical cohort

During the historical cohort period (2015–2019), several general measures for SSI prevention were already implemented at the participating centers. These included:

1. Enhanced Recovery After Surgery (ERAS) Protocols: The centers followed ERAS guidelines[5], including standard preoperative and perioperative measures to reduce complications, including SSIs.
2. Internal review of the incidence and risk factors for developing SSIs[6,7]
3. Assessment of long-term SAP in stented patients, which did not yield positive results[2]
4. Assessment of a new SAP based on piperacillin/tazobactam. All patients received piperacillin-tazobactam (4.5 g intravenously before skin incision, then 2.25 g every three hours until the end of surgery). The SAP showed promising results but was deemed impractical on a large scale.
5. Identification of the institutional microbiological flora and specific bacterial resistance to find a high-risk population that could benefit from tailored SAP[3]
6. Evaluation of rectal screening as a key tool for surveillance to reduce the in-hospital spread of MDR and treat colonized patients, emphasizing its relationship with bile cultures and postoperative outcomes[1]
7. Infection Control Measures: Standard infection control measures, such as proper skin preparation and sterile surgical techniques, were routinely followed.

© 2025 De Pastena M et al. *JAMA Network Open*.

8. Monitoring by infectious disease specialists: Although infectious disease specialists were involved in routine monitoring and postoperative antibiotic prescribing, there was no structured AMS program behind this.

Detailed implementation timeline

The creation of the dedicated AMS program for elective pancreatic surgery involved a multi-step process starting in 2015 and culminating in a structured intervention implemented beginning in January 2020. Here's a breakdown of the key phases:

1. *2015-2017: Review and data analysis phase:*
    - *Objective:* To understand the existing infectious outcomes and local microbiological landscape.
    - *Activities:*
        - Retrospective review of postoperative infectious outcomes (SSI rates, types of infections, etc.).
        - Analysis of center-specific microbiological data, including common pathogens and resistance patterns.
        - Identification of key areas for improvement in SAP and infection control practices.
    - *Output:* Identification of areas for improvement (based on data analysis).
2. *2017-2019: Development and protocol design phase:*
    - *Objective:* To design a multifaceted AMS program tailored to pancreatic surgery.
    - *Activities:*
        - Formation of a multidisciplinary team (surgeons, infectious disease specialists, microbiologists, pharmacists).
        - Development of specific protocols for preoperative rectal screening, intraoperative bile cultures, and tailored SAP selection.

        - Creation of educational materials for healthcare providers and patients.
        - Integration of the AMS program into the existing ERAS protocols[5].
    - *Output:* A finalized AMS protocol.
3. *January 2020: Implementation Phase:*
    - *Objective:* To formally launch the AMS program at all participating centers.
    - *Activities:*
        - Official launch of the AMS protocol.
        - Training sessions for healthcare providers on the new protocols.
        - Continuous monitoring of preoperative rectal screening and intraoperative bile cultures.
        - Continuous monitoring of SAP appropriateness and SSI rates.
        - Data Collection.
4. *2020-2022: Ongoing Monitoring and Refinement:*
    - *Objective:* To continuously monitor the effectiveness of the AMS program and make necessary adjustments based on ongoing data analysis.
    - *Activities:*
        - Regular review of SAP appropriateness and SSI rates.
        - Audits of antibiotic usage.
        - Discussions among the multidisciplinary team to identify areas for improvement.
        - Adjustments to the AMS protocol as needed based on new data and feedback.

eMethods 2

CDC SSI Definition and Classification[8]:

 Surgical Site Infections (SSIs) are defined as an infectious process localized to the level of the surgical incision and are classified as either incisional or organ/space. Incisional SSI is further divided into those that involve only the skin and subcutaneous tissue (superficial incisional SSI) and those that involve the deeper soft tissues of the incision (deep incisional SSI). The presence of bacteria or fungi in a sample from patients colonized by MDR bacteria is considered indicative of an SSI if associated with a septic syndrome (as defined by ACCP/SCCM 2001 International sepsis definitions criteria), with imaging (CT or MRI) suggestive of infected abdominal collections, and with significant modifications in biomarkers (i.e., high levels of C-reactive protein, procalcitonin, white blood cell count) in order to differentiate real infections from mere colonization.

- Superficial SSI:

A superficial surgical site infection (SSI) is defined as an infection that:

- Occurs within 30 days after the operative procedure
  AND
- Involves only the skin or subcutaneous tissue of the incision
  AND
- At least one of the following is present:
  1. Purulent drainage from the superficial incision.
  2. Organisms isolated from an aseptically obtained culture of fluid or tissue from the superficial incision.
  3. At least one of the following signs or symptoms: pain or tenderness, localized swelling, redness, or heatAND the superficial incision is deliberately opened by a surgeon (unless the incision is culture-negative).
  4. Diagnosis of a superficial SSI by the surgeon or attending physician.

- Deep SSI:

A deep surgical site infection (deep SSI), according to the CDC (Centers for Disease Control and Prevention), is defined as an infection that:

© 2025 De Pastena M et al. *JAMA Network Open*.

- Occurs within 30 days of the operative procedure if no implant is in place, or within 90 days if an implant is left in place and the infection appears related to the operative procedure
  AND
- Involves deep soft tissues of the incision (e.g., fascia and muscle layers)
  AND
- The infection meets at least one of the following criteria:

1. Purulent drainage from the deep incision but not from the organ/space component.
2. A deep incision that spontaneously dehisces or is deliberately opened by a surgeon when the patient has at least one of the following signs or symptoms:
   - Fever (>38°C)
   - Localized pain or tenderness,
     unless the incision is culture-negative.
3. An abscess or other evidence of infection involving the deep incision found on direct examination, during reoperation, or by histopathologic or radiologic examination.
4. Diagnosis of a deep incisional SSI by a surgeon or attending physician.

If the infection involves both superficial and deep incision sites, it is classified as deep. If it extends into the organ/space, it may be considered an organ/space SSI.

- Organ/Space SSI:

- Occurs within 30 days after the operative procedure if no implant is in place, or within 90 days if an implant is left in place and the infection appears related to the operative procedure
  AND
- Involves any part of the anatomy (e.g., organs or spaces) other than the incision, which was opened or manipulated during the surgical procedure
  AND
- The infection meets at least one of the following criteria:

1. Purulent drainage from a drain placed into the organ/space.
2. Organisms isolated from an aseptically obtained culture of fluid or tissue in the organ/space.
3. An abscess or other evidence of infection involving the organ/space that is seen during direct examination, reoperation, or by histopathologic or radiologic examination.
4. Diagnosis of an organ/space SSI by a surgeon or attending physician.

 Surgical Antibiotic Prophylaxis (SAP) Appropriateness: Surgical antibiotic prophylaxis entails administering antibiotics before surgery to prevent infections. Assessing the appropriateness of SAP determines whether the selected antibiotics effectively target the bacteria likely to cause infections in that particular surgical procedure. To evaluate SAP appropriateness, the antibiogram of

© 2025 De Pastena M et al. *JAMA Network Open*.

the isolated bacteria is utilized to identify their sensitivity or resistance to the antibiotics. Appropriateness and coverage can be considered synonyms.

Clavien-Dindo classification of complications (major complications are graded ≥3, from Clavien PA et al.,[9])

Grade I

Any deviation from the normal postoperative course without the need for pharmacological treatment or surgical, endoscopic, or radiological interventions.
Allowed treatments include:
Antiemetics
Antipyretics
Analgesics
Diuretics
Electrolytes
Physiotherapy
Bedside wound opening

Grade II

Requires pharmacological treatment with drugs other than those allowed for Grade I.
Includes:
Blood transfusions
Total parenteral nutrition
Antibiotics for infection

Grade III

IIIa: Intervention without general anesthesia (e.g., drain placement, endoscopy).

IIIb: Intervention under general anesthesia (e.g., reoperation, major procedures).

Grade IV

Life-threatening complications requiring ICU management.

IVa: Single-organ dysfunction (e.g., respiratory failure requiring ventilation).

IVb: Multi-organ dysfunction.

Grade V

Death of the patient due to complication.

International Study Group of Pancreatic surgery (ISGPS) grading of postoperative pancreatic fistula (POPF, from Bassi C et al., [10])

- Biochemical Leak (Formerly Grade A): Amylase-rich fluid in the drain with no clinical impact
- *Clinically Relevant POPF (Grades B & C)*
  Grade B:
    - Prolonged drainage

- Antibiotics
- Percutaneous drainage
- Delay in diet resumption or discharge
- No organ failure or major reoperation

Grade C:

- Major reoperation
- Organ failure
- May result in death

International Study Group of Pancreatic Surgery (ISGPS) grading of delayed gastric emptying (DGE, from Wente MN et al.[11])

- Grade A: the need for a nasogastric tube (NGT) between postoperative days (POD) 4 and 7, reinsertion of the NGT due to nausea/vomiting by POD 4, or inability to tolerate a solid or semi-solid diet between POD 7 and 13.

- Grade B: the need for a nasogastric tube (NGT) between post-operative days (POD) 8 and 14, or reinsertion of the NGT after POD 7, or the inability to tolerate a solid or semi-solid diet between POD 14 and 20.

- Grade C: the need for a nasogastric tube (NGT) for more than 14 days or needs reinsertion of the NGT after day 14, and/or cannot tolerate unlimited oral intake by postoperative day 21.

International Study Group of Pancreatic Surgery (ISGPS) grading of post-pancreatectomy hemorrhage (PPH, from Wente MN et al.[12])

- Grade A: a mild bleeding event that:
  - Occurs early (within the first 24 hours after surgery),
  - OR is classified as late (after 24 hours), but
  - Has minimal clinical impact.

- Grade B: clinically significant bleeding that:
  - Occurs either early (‚â§24 hours) or late (>24 hours) after surgery

  AND
  - Requires intervention beyond just monitoring, but does not cause life-threatening instability.

- Grade C: severe, often life-threatening bleeding event that:
  - Can occur early (‚â§24 hours) or late (>24 hours) after surgery

  AND
  - Requires major intervention, such as:
  - Emergency surgery (reoperation)
  - Angiographic embolization
  - ICU management for hemodynamic instability

© 2025 De Pastena M et al. *JAMA Network Open*.

International Study Group of Pancreatic Surgery (ISGPS) grading of chyle leak (from Besselink M et al.[13])

- Grade A:
  - No specific intervention other than oral dietary restrictions; grade B, prolongation of hospital stay, nasoenteral nutrition with dietary restriction, total parenteral nutrition, octreotide, maintenance of surgical drains, or placement of new percutaneous drains; and grade C,
- Grade B:
  - Prolongation of hospital stay, nasoenteral nutrition with dietary restriction, total parenteral nutrition, octreotide, maintenance of surgical drains, or placement of new percutaneous drains
- Grade C:
  - Need for other more invasive in-hospital treatment, intensive care unit admission, or mortality

International Study Group of Liver Surgery (ISGLS) definition and grading of bile leak (from Koch M, et al.[14])

- Grade A: a bile leak that has little or no impact on the patient's clinical management. It typically involves a transient leak that can be managed with intra-abdominal drains, and often resolves without requiring additional diagnostic or therapeutic interventions.

- Grade B: a bile leakage that requires a change in the patient's clinical management, but can be treated without requiring re-laparotomy (reopening the abdomen). This means a Grade B leak necessitates some form of intervention, such as endoscopic or percutaneous drainage, but doesn't escalate to the severity of needing a surgical procedure to fix it.

- Grade C: severe bile leak that requires a re-operation for treatment, usually due to bile peritonitis. This is the most severe grade of bile leak, indicating a significant complication requiring surgical intervention.

© 2025 De Pastena M et al. *JAMA Network Open*.

eMethods 3

Rectal screening

The universal rectal screening was performed at least two weeks before surgery. The swab was streaked on selective media ChromoID® ESBL (bioMérieux, Lyon, France) plus an ertapenem disk (10µg), and MacConkey agar plus a meropenem disk (10µg) was used to detect ESBL-producing Enterobacterales, CPE and *P. aeruginosa.* CNA agar plus 6 mg/ml of vancomycin was used to detect Vancomycin-Resistant Enterococci (VRE). The strains were identified using Maldi-tof technology with VITEK MS® (bioMérieux, Lyon, France). Carbapenemase production was confirmed with CarbaNP rapid test[1], while ESBL production was confirmed with ESBL NDP rapid test[7]. The antimicrobial susceptibility testing was performed by the VITEK-2® automated system (bioMérieux, Lyon, France). Results were interpreted according to the latest EUCAST guidelines[8]. Glycopeptides resistance for enterococci was confirmed by Etest (bioMérieux, Lyon, France).

© 2025 De Pastena M et al. *JAMA Network Open*.

eFigure 1. Propensity Score Weighting Covariate Balance

Panel A: Standardized mean differences (SMDs) and Kolmogorov–Smirnov (KS) statistics for all baseline covariates after weighting.

Panel B: Covariate balance plot showing Spearman correlations between treatment assignment and covariates before (red) and after (blue) weighting. Post-weighting correlations are centered around zero, confirming improved covariate balance.

Panel A Balance Metrics

Covariate	SMD	KS Statistic
**Age**	0.0051	0.0060
**Neoadjuvant Therapy**	0.0039	0.0018
**Type of drain: No**	0.0009	0.0002
**Type of drain: Plastic**	0.0032	0.0013
**Type of drain: Metallic**	0.0092	0.0024
**Type of drain: PTBD**	-0.0081	0.0039
**Operation time**	-0.0053	0.0035
**Surgical Approach: Open**	-0.0024	0.0008
**Surgical Approach: Laparoscopic**	-0.0075	0.0016
**Surgical Approach: Robotic**	0.0096	0.0024
**Estimated Blood loss**	-0.0006	0.0062
**Pathology: PDAC**	0.0022	0.0011
**Pathology: pNET**	-0.0004	0.0001
**Pathology: IPMN**	0.0008	0.0002
**Pathology: other cysts**	0.0100	0.0019
**Pathology: other periampullary cancers**	-0.0035	0.0010
**Pathology: Other**	-0.0066	0.0020
**BMI**	0.0087	0.0054
**CACI**	0.0018	0.0009
**ASA**	-0.0049	0.0021
**Rectal Screening No**	-0.0055	0.0022
**Rectal Screening Yes**	0.0009	0.0003
**Rectal Screening Undefined**	0.0070	0.0019
**Type of surgery: Pancreaticoduodenectomy**	-0.0048	0.0024
**Type of Surgery: Left Pancreatectomy**	0.0092	0.0042
**Type of Surgery: Total Pancreatectomy**	-0.0056	0.0018
**Gender (Female)**	-0.0062	0.0031
**Jaundice No**	-0.0147	0.0067
**Jaundice Yes**	0.0052	0.0026
**Jaundice Undefined**	0.0092	0.0042

Panel B Balance Plot

© 2025 De Pastena M et al. *JAMA Network Open*.

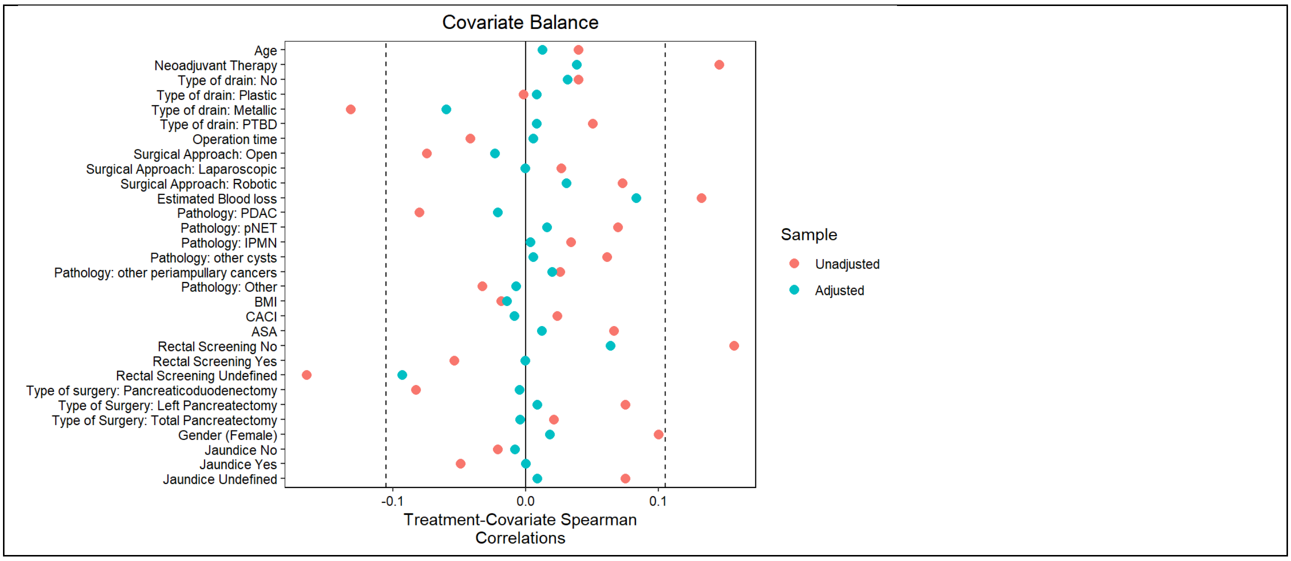

BMI: body mass index; CACI: Charlson Comorbidity Index; MDR: multi-drug resistant bacteria ASA: American Society of Anesthesiologists

© 2025 De Pastena M et al. *JAMA Network Open*.

eFigure 2. Study Flowchart

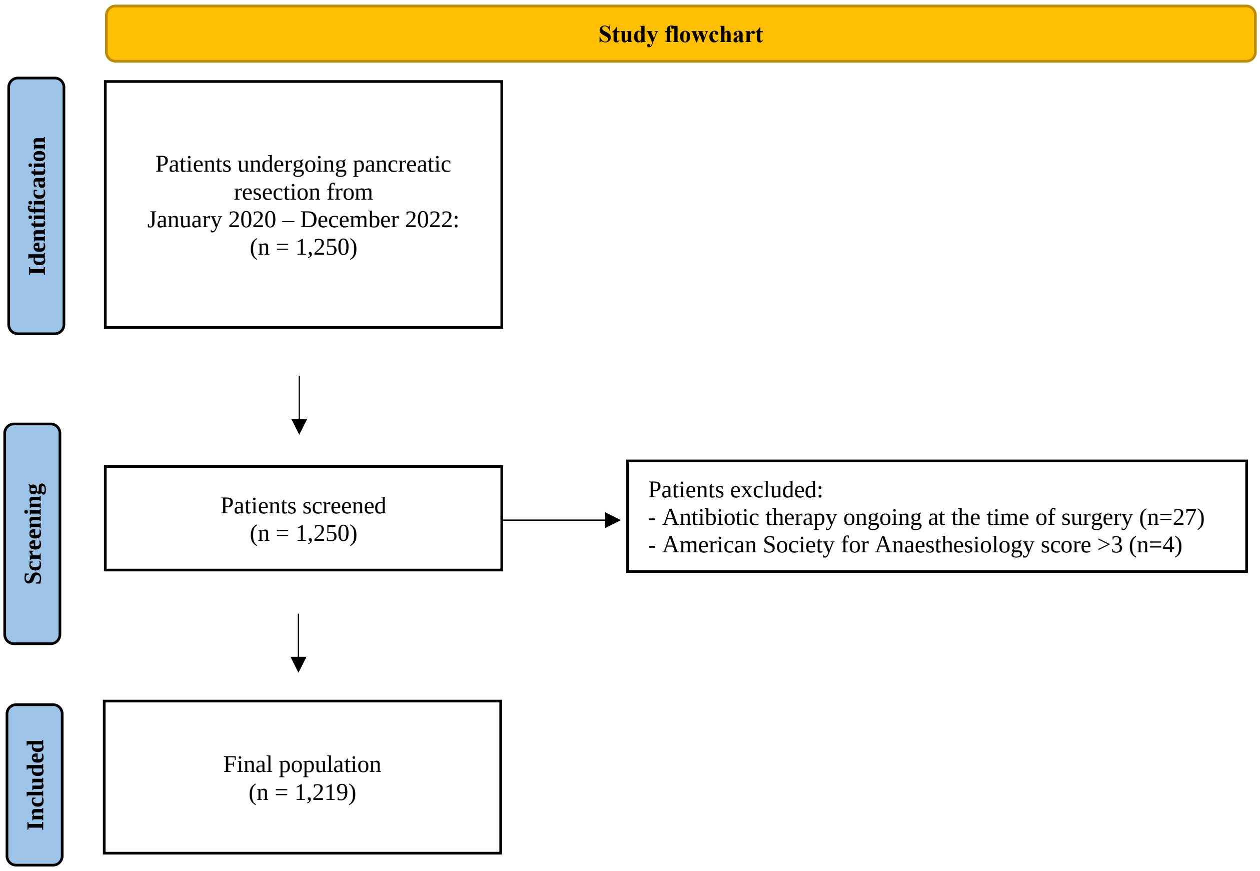

eFigure 3. Microbiological Report of Superficial SSI

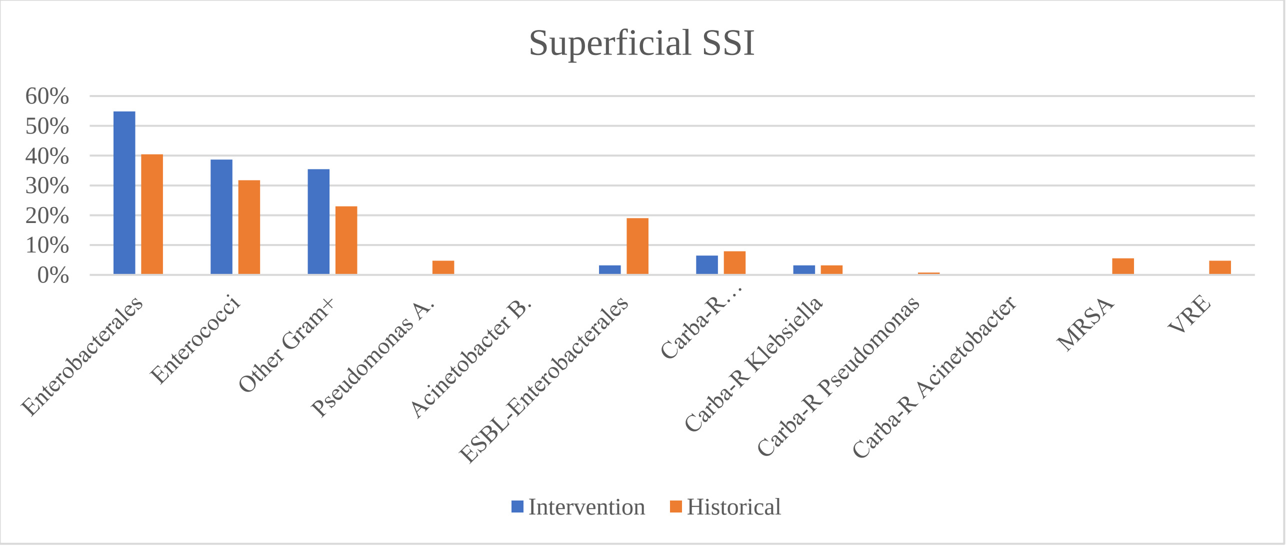

Pseudomonas A.: Pseudomonas Aeruginosa; Carba-R Enterobacterales: Carbapenem-Resistant Enterobacterales; Carba-R Klebsiella: Carbapenem-Resistant Klebsiella spp.; Carba-R Pseudomonas: Carbapenem-Resistant Pseudomonas Aeruginosa; Carba-R Acinetobacter: Carbapenem-Resistant Acinetobacter Baumanii; MRSA: Methicillin-Resistant Staphylococcus Aureus; VRE: Vancomycin-Resistant Enterococci.

© 2025 De Pastena M et al. *JAMA Network Open*.

eFigure 4. Microbiological Report of Deep SSI

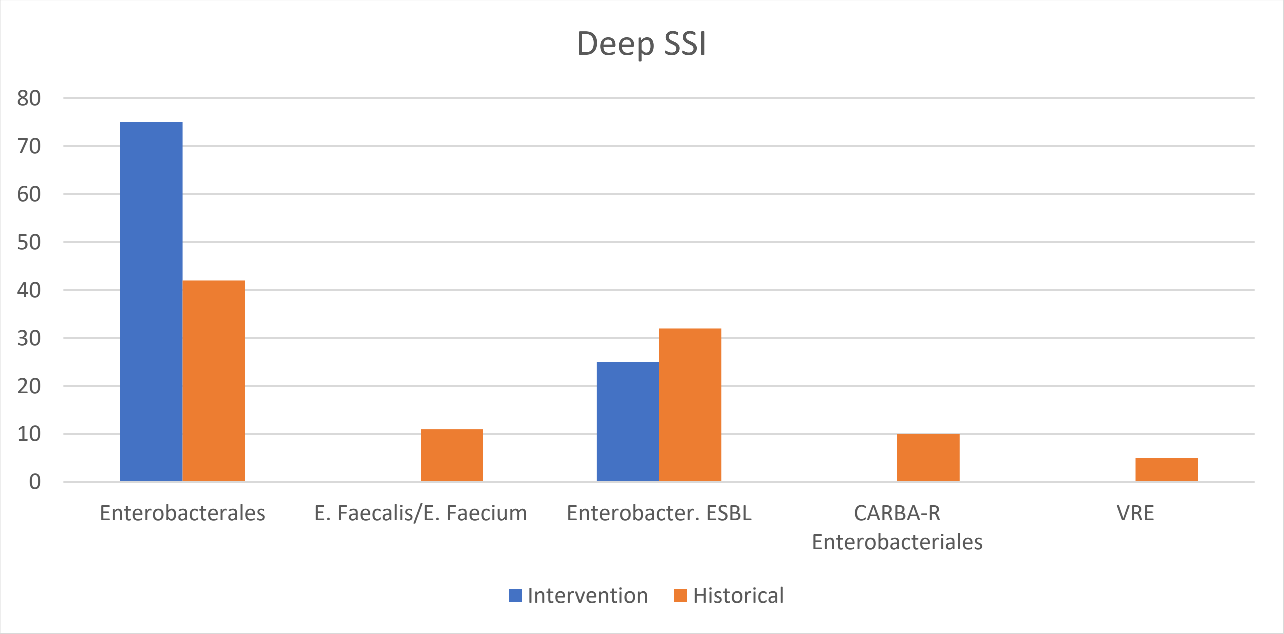

Carba-R: Carbapenem-Resistant; VRE: Vancomycin-Resistant Enterococci.

eFigure 5. Microbiological Report of Organ/Space SSI

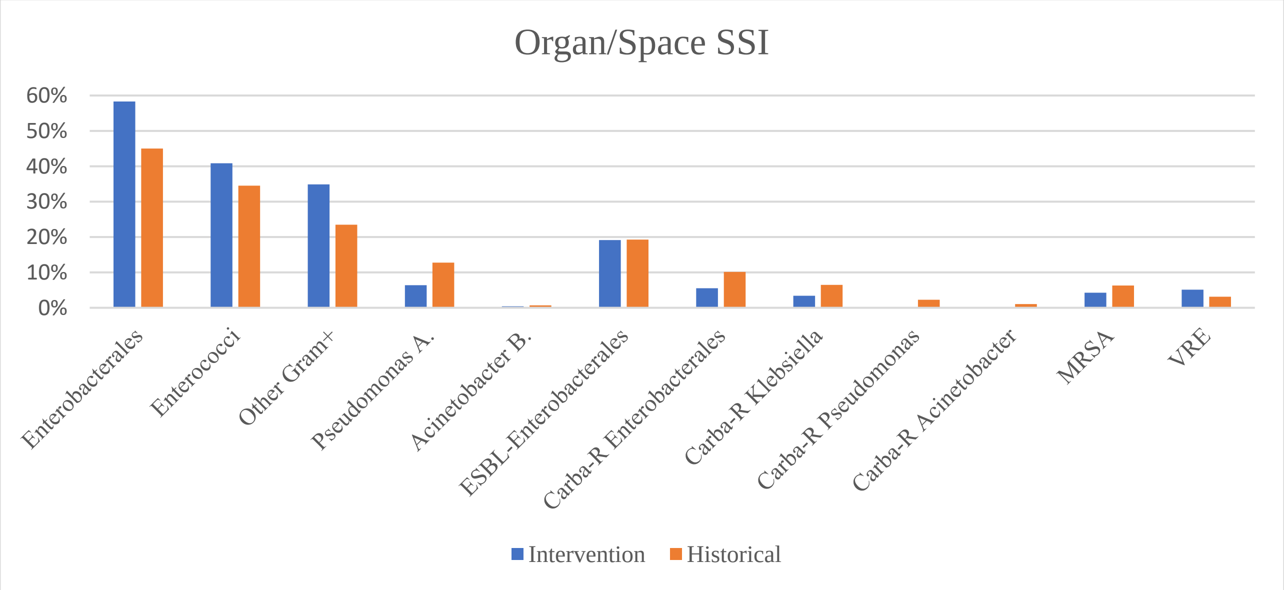

Pseudomonas A.: Pseudomonas Aeruginosa; Carba-R Enterobacterales: Carbapenem-Resistant Enterobacterales; Carba-R Klebsiella: Carbapenem-Resistant Klebsiella spp.; Carba-R Pseudomonas: Carbapenem-Resistant Pseudomonas Aeruginosa; Carba-R Acinetobacter: Carbapenem-Resistant Acinetobacter Baumanii; MRSA: Methicillin-Resistant Staphylococcus Aureus; VRE: Vancomycin-Resistant Enterococci.

© 2025 De Pastena M et al. *JAMA Network Open*.

eTable 2. The Primary Outcome Through Center-Specific Analyses

	Historical cohort	Intervention cohort
SSI, n (%)	653 (30.1)	251 (20.6)
Verona, n (%)	574 (31.2)	234 (22)
Roma, n (%)	39 (26.4)	12 (14.6)
Firenze, n (%)	40 (22)	5 (6.9)
Superficial, n (%)	126 (5.8)	31 (2.5)
Verona, n (%)	101 (5.5)	28 (2.6)
Roma, n (%)	12 (8.1)	2 (2.4)
Firenze, n (%)	13 (7.1)	1 (1.4)
Deep, n (%)	19 (0.9)	4 (0.3)
Verona, n (%)	10 (0.5)	3 (0.3)
Roma, n (%)	3 (2.0)	1 (1.2)
Firenze, n (%)	6 (3.3)	0 (0)
Organ/space, n (%)	571 (26.3)	235 (19.3)
Verona, n (%)	519 (28.2)	222 (20.8)
Roma, n (%)	29 (19.6)	9 (11)
Firenze, n (%)	23 (12.6)	4 (5.6)

SSI: surgical site infection.

© 2025 De Pastena M et al. *JAMA Network Open*.

eTable 3. Primary and Secondary Outcomes, Including Only Formal Pancreatic Resections

	**Historical cohort, n=2168**	**Intervention cohort, n=1094**	**P-Value**
SSI, n (%)	653 (30.1)	240 (21.9)	**<0.001**
Superficial, n (%)	126 (5.8)	29 (2.7)	**<0.001**
Deep, n (%)	19 (0.9)	4 (0.4)	0.072
Organ/space, n (%)	571 (26.3)	226 (20.7)	**<0.001**
Any complications, n (%)	1260 (58.1)	565 (51.6)	**<0.001**
Major complications*, n (%)	433 (20.0)	192 (15.8)	**0.002**
HAI (any type), n (%)	879 (40.5)	352 (32.2)	**<0.001**
Postoperative pancreatic fistula, n (%)			**<0.001**
Grade B	314 (16.4)	181 (19)	
Grade C	79 (4.1)	17 (1.8)	
Biliary fistula (any grade), n (%)	114 (5.3)	80 (7.3)	0.1
Chyle leak (any grade), n (%)	97 (4.5)	47 (4.3)	0.4
Enteric fistula (any grade), n (%)	56 (2.6)	27 (2.5)	0.5
Post-pancreatectomy hemorrhage (any grade), n (%)	306 (14.1)	122 (11.2)	**0.010**
Delayed gastric emptying (any grade), n (%)	282 (13.0)	122 (11.2)	0.071
Intensive care unit admission (any grade), n (%)	262 (12.1)	118 (10.8)	0.1
Reoperation, n (%)	179 (8.3)	79 (7.2)	0.2
Length of stay, days, median (IQR)	10 [7-18]	8 [7-18]	**0.031**
Mortality, n (%)	70 (3.2)	33 (3)	0.4

SSI: surgical site infection; ESBL: Extended-spectrum beta-lactamases-producer microbe; Carba-R: carbapenem-resistant Enterobacterales; VRE: Vancomycin-resistant Enterococci.
Values in bold are statistically significant.

© 2025 De Pastena M et al. *JAMA Network Open*.

eFigure 6. Estimated Probabilities of SSI

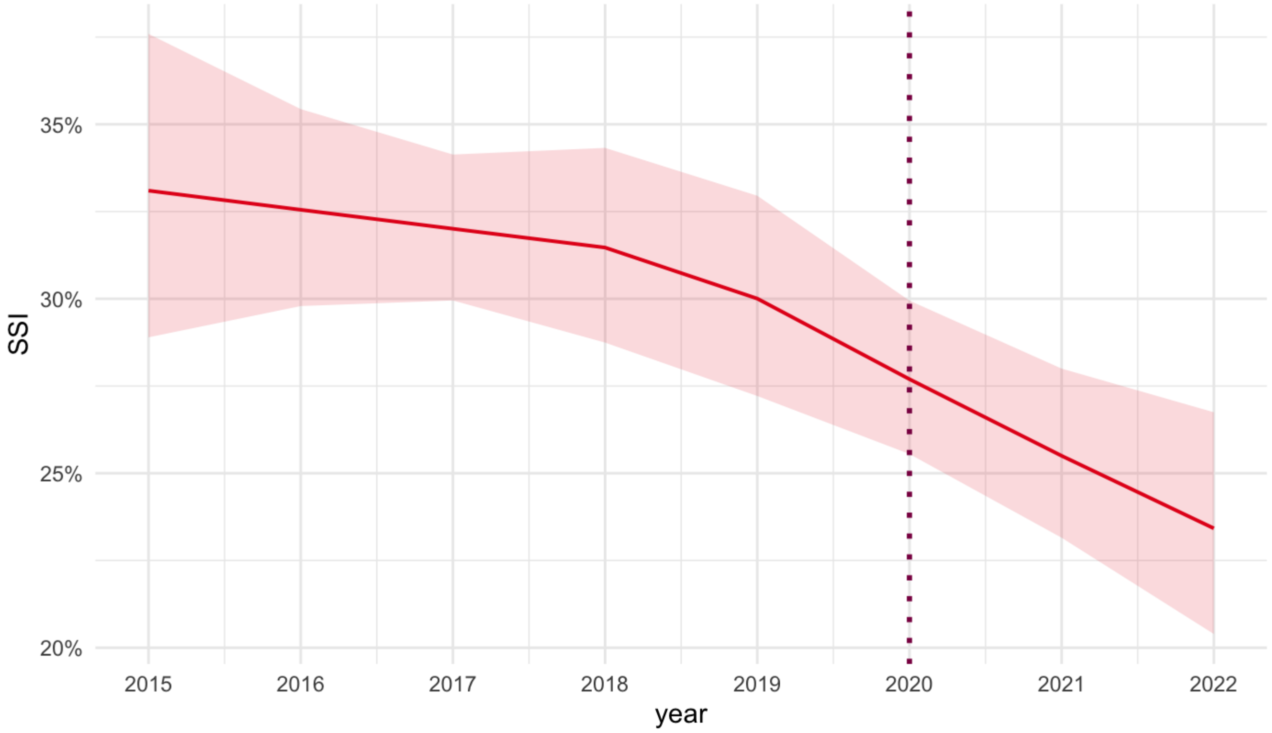

The dotted line indicates the introduction of the AMS

© 2025 De Pastena M et al. *JAMA Network Open*.

eReferences

1. De Pastena M, Paiella S, Azzini AM, et al. Preoperative surveillance rectal swab is associated with an increased risk of infectious complications in pancreaticoduodenectomy and directs antimicrobial prophylaxis: an antibiotic stewardship strategy? *HPB (Oxford)* 2018; **20**(6): 555-62.
2. De Pastena M, Paiella S, Marchegiani G, et al. Postoperative infections represent a major determinant of outcome after pancreaticoduodenectomy: Results from a high-volume center. *Surgery* 2017; **162**(4): 792-801.
3. Fong ZV, McMillan MT, Marchegiani G, et al. Discordance Between Perioperative Antibiotic Prophylaxis and Wound Infection Cultures in Patients Undergoing Pancreaticoduodenectomy. *JAMA Surg* 2016; **151**(5): 432-9.
4. De Pastena M, Paiella S, Azzini AM, et al. Antibiotic Prophylaxis with Piperacillin-Tazobactam Reduces Post-Operative Infectious Complication after Pancreatic Surgery: An Interventional, Non-Randomized Study. *Surg Infect (Larchmt)* 2021; **22**(5): 536-42.
5. Melloul E, Lassen K, Roulin D, et al. Guidelines for Perioperative Care for Pancreatoduodenectomy: Enhanced Recovery After Surgery (ERAS) Recommendations 2019. *World J Surg* 2020; **44**(7): 2056-84.
6. Costi R, De Pastena M, Malleo G, et al. Poor Results of Pancreatoduodenectomy in High-Risk Patients with Endoscopic Stent and Bile Colonization are Associated with E. coli, Diabetes and Advanced Age. *J Gastrointest Surg* 2016; **20**(7): 1359-67.
7. De Pastena M, Marchegiani G, Paiella S, et al. Impact of preoperative biliary drainage on postoperative outcome after pancreaticoduodenectomy: An analysis of 1500 consecutive cases. *Dig Endosc* 2018; **30**(6): 777-84.
8. Berrios-Torres SI, Umscheid CA, Bratzler DW, et al. Centers for Disease Control and Prevention Guideline for the Prevention of Surgical Site Infection, 2017. *JAMA Surg* 2017; **152**(8): 784-91.
9. Dindo D, Demartines N, Clavien PA. Classification of surgical complications: a new proposal with evaluation in a cohort of 6336 patients and results of a survey. *Ann Surg* 2004; **240**(2): 205-13.
10. Bassi C, Marchegiani G, Dervenis C, et al. The 2016 update of the International Study Group (ISGPS) definition and grading of postoperative pancreatic fistula: 11 Years After. *Surgery* 2017; **161**(3): 584-91.
11. Wente MN, Bassi C, Dervenis C, et al. Delayed gastric emptying (DGE) after pancreatic surgery: a suggested definition by the International Study Group of Pancreatic Surgery (ISGPS). *Surgery* 2007; **142**(5): 761-8.
12. Wente MN, Veit JA, Bassi C, et al. Postpancreatectomy hemorrhage (PPH): an International Study Group of Pancreatic Surgery (ISGPS) definition. *Surgery* 2007; **142**(1): 20-5.
13. Besselink MG, van Rijssen LB, Bassi C, et al. Definition and classification of chyle leak after pancreatic operation: A consensus statement by the International Study Group on Pancreatic Surgery. *Surgery* 2017; **161**(2): 365-72.
14. Koch M, Garden OJ, Padbury R, et al. Bile leakage after hepatobiliary and pancreatic surgery: a definition and grading of severity by the International Study Group of Liver Surgery. *Surgery* 2011; **149**(5): 680-8.

© 2025 De Pastena M et al. *JAMA Network Open*.
