# Supplementary material for: An Antibiotic Stewardship Program in Pancreatic Surgery
Source: JAMA Netw Open. 2025 Jul 11;8(7):e2520149. doi: 10.1001/jamanetworkopen.2025.20149 (PMC12254888; doi:10.1001/jamanetworkopen.2025.20149)
Supplement: Supplement 2. — Data Sharing Statement [file jamanetwopen-e2520149-s002.pdf]

Data Sharing Statement

De Pastena. An Antibiotic Stewardship Program in Pancreatic Surgery. *JAMA Netw Open*. Published July 11, 2025. doi:10.1001/jamanetworkopen.2025.20149

Data
Data available: No
